# Comparative effectiveness of bariatric surgeries in patients with obesity and type 2 diabetes mellitus: A network meta‐analysis of randomized controlled trials

**DOI:** 10.1111/obr.13030

**Published:** 2020-04-14

**Authors:** Li Ding, Yuxin Fan, Hui Li, Yalan Zhang, Dongwang Qi, Shaofang Tang, Jingqiu Cui, Qing He, Chuanjun Zhuo, Ming Liu

**Affiliations:** ^1^ Department of Endocrinology and Metabolism Tianjin Medical University General Hospital Tianjin China; ^2^ Department of School of Mental Health Jining Medical University Jining China

**Keywords:** bariatric surgeries, diabetes remission, cardiometabolic outcome, network meta‐analysis

## Abstract

A network meta‐analysis of randomized controlled trials (RCTs) was performed to determine the hierarchies of different bariatric surgeries in patients with obesity and type 2 diabetes mellitus (T2DM), in terms of diabetes remission and cardiometabolic outcomes. Seventeen RCTs and six bariatric surgeries, including single anastomosis (mini) gastric bypass (mini‐GBP), biliopancreatic diversion without duodenal switch (BPD), laparoscopic‐adjustable gastric banding (LAGB), laparoscopic sleeve gastrectomy (LSG), Roux‐en‐Y gastric bypass (RYGBP), greater curvature plication (GCP) and nonsurgical treatments (NST) were included. Mini‐GBP, BPD, LSG, RYGBP and LAGB (from best to worst), as compared with NST, were all significantly associated with the remission of T2DM. For the follow‐up period > 3 years, BPD, mini‐GBP, RYGBP and LSG (from best to worst) were significantly superior to NST in achieving the remission of T2DM. For secondary outcomes, the overall ranking for bariatric surgeries was RYGBP > BPD > LSG > LAGB after comprehensively weighting glucose, weight, systolic and diastolic pressure, total cholesterol, triglycerides, high‐density lipoprotein cholesterol (HDL‐C) and low‐density lipoprotein cholesterol (LDL‐C). Mini‐GBP has the greatest probability of achieving diabetes remission in adults with obesity and T2DM, yet BPD was the most effective in long‐term diabetes remission. RYGBP appears to be the most favourable alternative treatment to manage patients with cardiometabolic conditions.

## 1 INTRODUCTION

Type 2 diabetes mellitus (T2DM) is characterized by insulin resistance caused by pancreatic β‐cell dysfunction.^1^ From 1980 to 2004, the incidence and prevalence of T2DM nearly quadrupled owing to the global rise in obesity, sedentary lifestyles and population aging.^2^ As the eighth leading cause of disability in 2016, diabetes substantially contributes to the socio‐economic pressures of individuals and the overwhelming health care costs.^3^ More than 60% of patients with T2DM have a body mass index (BMI) ≥ 30 kg/m^2^, and patients with obesity are more likely to develop T2DM.^4^ Whole‐genome analysis of gene expression products (i.e. mRNAs) has uncovered several genetic associations between T2DM and obesity by correlating genotypes with phenotypes.^5^ Sustained weight loss is a highly effective strategy to treat and prevent T2DM, with low‐calorie diets^6^ and bariatric surgeries^7^ being the ideal treatments to achieve T2DM remission.

The primary purpose of bariatric surgery is to achieve and sustain significant weight loss, which leads to the improvement and remission of many obesity‐related comorbidities, especially T2DM.^8^, ^9^, ^10^ Considerable evidence has suggested that T2DM can be controlled by bariatric surgery in patients with morbid obesity.^11^, ^12^, ^13^, ^14^ In addition to weight loss, bariatric surgery provides additional health benefits, including improvements in cardiometabolic comorbidities like dyslipidaemia, hypertension and obstructive sleep apnoea.^15^ Two previous network meta‐analyses attempted to construct a clear hierarchy showing the effectiveness of different bariatric surgeries for T2DM.^16^, ^17^ However, their results were based on studies not specific to patients with obesity and T2DM and failed to comprehensively consider the effects of cardiometabolic conditions. To provide concrete evidence for clinical practice, there is an urgent need for a thorough comparison of diabetes remission and cardiometabolic outcomes. Herein, we performed a network meta‐analysis to compare the effectiveness of different bariatric surgeries focusing on their ability to achieve diabetes remission and their effects on cardiometabolic outcomes in patients with obesity and T2DM.

## 2 METHODS

### 2.1 Literature search

This network meta‐analysis was conducted in compliance with the PRISMA Extension Statement for Reporting of Systematic Reviews Incorporating Network Meta‐analyses of Health Care Interventions: Checklist and Explanations.^18^ The study protocol was prespecified and registered on the International Prospective Register of Systematic Reviews (PROSPERO) under the code CRD42018110775.^19^ A literature search was conducted on Medline through PubMed, Embase and Web of Knowledge in July 2019. The references of articles identified through the initial screening were used to identify articles missed by the computerized database search. The following search terms were used: bariatric surgery, bariatric operation, bariatric procedure, obesity surgery, metabolic surgery, stomach stapling, biliopancreatic bypass, biliopancreatic diversion, duodenal switch, sleeve gastrectomy and gastric bypass. These terms were combined using the set operator “AND” with type 2 diabetes mellitus, non‐insulin‐dependent diabetes (NIDDM) and T2DM.

### 2.2 Eligibility and exclusion criteria

The eligibility criteria were as follows. Participants: Adults (16 years or older) who were overweight or obese with clearly documented T2DM (BMI ≥ 30 kg/m^2^ or BMI ≥ 25 kg/m^2^ with at least one obesity‐related comorbidity, including T2DM). Intervention: A history of at least one bariatric surgery, such as biliopancreatic diversion without duodenal switch (BPD), Roux‐en‐Y gastric bypass (RYGBP), single anastomosis (mini) gastric bypass (mini‐GBP), laparoscopic‐adjustable gastric banding (LAGB), laparoscopic sleeve gastrectomy (LSG) or greater curvature plication (GCP). Comparators: Nonsurgical treatments (NST) or another bariatric surgery. Outcome: The primary outcomes were complete T2DM remission, defined as HbA1c levels < 6.0% at consecutive annual clinic visits with no use of anti‐hyperglycaemic medications^20^ or as defined by the individual studies. Secondary outcomes were cardiometabolic related, including mean changes in weight loss, blood pressure, total cholesterol, triglycerides, low‐density lipoprotein cholesterol (LDL‐C) and high‐density lipoprotein cholesterol (HDL‐C). Study design: The randomized controlled trials (RCTs) were targeted towards adult patients with obesity and T2DM.

The exclusion criteria were as follows: (1) patients without T2DM; (2) non‐RCTs, reviews or reports solely focusing on laboratory findings; (3) trials published only as abstracts; (4) animal‐only experiments; and (5) studies reported in a language other than English.

### 2.3 Study selection and data extraction

Two investigators independently reviewed the titles and abstracts of the articles identified with the search criteria. Multiple publications of the same study were identified and grouped together as ‘kinned' citations, which were counted only once to avoid double‐counting of patients. The parent study was normally the latest publication. The full‐text versions of potentially eligible studies were then assessed. A predefined data extraction sheet, which was designed according to the Cochrane Handbook,^21^ was used to extract relevant information, including author information, study design, participant characteristics, interventions, outcome measures, study duration and other information as needed. Authors were directly contacted to seek additional information in cases where the data were unclear or not reported. Any discrepancies were resolved by consensus and arbitration by other investigators in the review team.

### 2.4 Risk of bias assessment

Two independent reviewers assessed the risk of bias of all included studies according to the Cochrane Handbook.^21^ Studies were classified to have a high, low or unclear risk of bias based on the adequacy of sequence generation, allocation concealment, blinding of participants and personnel, blinding of outcome assessment, method of addressing incomplete data, selective reporting and other potential biases. Disagreements were resolved first by discussion, followed by consulting with a third arbitrator if needed. Graphic representations of potential biases within and across the studies were generated using RevMan V.5.1 (Cochrane, London, UK).

### 2.5 Statistical analysis

We calculated odds ratios (ORs) and standardized mean differences (SMDs) with their 95% credible intervals (CrIs) for the dichotomous and continuous outcomes of this network analysis. The statistical heterogeneity was estimated using *I* ^2^ statistics, which describe the percentage of variability across studies caused by heterogeneity rather than chance.^22^ Subgroup analyses were performed to identify potential moderators of the effects on outcomes. Publication bias was evaluated visually using funnel plots, which summarized pooled treatment comparisons and comparison‐adjusted funnel plots for small‐study effects. Inconsistency checks were performed for closed loops in the network,^23^ assuming there was a common heterogeneity parameter across all the loops in the system, as derived from the network meta‐analysis model. To rank the bariatric surgeries, we calculated the probabilities of the surface under the cumulative ranking curve (SUCRA). In the network meta‐analysis, zero cells were corrected with the command “network setup” in Stata. The frequentist‐based network meta‐analysis was performed using Stata V.14 (Stata, College Station, TX, USA). All statistical differences were considered significant when *P* was less than 0.05.

### 2.6 Patient and public involvement

There was no patient and public involvement as this was a network meta‐analysis.

## 3 RESULTS

### 3.1 Characteristics of included studies

Seventy RCTs involving 1108 adult subjects were included in this network meta‐analysis (Figure S1).^13^, ^24^, ^25^, ^26^, ^27^, ^28^, ^29^, ^30^, ^31^, ^32^, ^33^, ^34^, ^35^, ^36^, ^37^, ^38^, ^39^ The features of the studies are summarized in Table 1. Although there is substantial variation in terms of treatment strategies, surgical techniques and publication years, the baseline patients' characteristics were similar among all the interventions. The earliest study was conducted in 2008, while the latest one was in 2018. The duration of the trials ranged from 0.5 to 5 years, and seven studies had follow‐up periods greater than 3 years. The interventions included six bariatric surgeries (BPD, GCP, LAGB, LSG, RYGBP and mini‐GBP) and NST. Most patients were older than 30. Six studies were reported as unblinded or open‐label clinical trials,^13^, ^24^, ^28^, ^31^, ^36^, ^38^ while three studies reported that the study investigators were aware of treatment allocations, as shown in Figures S2 and S3. A graphical network structure shows the network of trials for different primary and secondary outcomes (Figure S4A‐J).

**TABLE 1:** Baseline characteristics of the included studies

Study	Country	Follow‐up (years)	No. of patients	Age	Men, *n* (%)	BMI, kg/m^2^	Systolic pressure	Diastolic pressure	Plasma glucose	Total cholesterol	Triglycerides	HDL‐C	LDL‐C
Dixon 2008	Australia	2	30	46.6 (7.4)	15 (50)	37.0 (2.7)	136.4 (15.6)	86.6 (9.4)	156.7 (38.5)	201.8 (32.7)	190.6 (106.6)	47.1 (10.1)	
			30	47.1 (8.7)	13 (43)	37.2 (2.5)	135.3 (14.4)	84.5 (9.8)	158.0 (48.7)	198.2 (56.7)	188.7 (111.8)	48.1 (11.1)	
Liang 2013	China	1	31	50.81 (5.44)	22 (71.0)	30.48 (0.94)	160.81 (7.77)	88.58 (5.53)	169.2 (20.88)	93.78 (9.9)	61.02 (21.24)	16.02 (3.06)	69.12 (11.34)
			36	51.75 (6.70)	24 (66.7)	30.34 (1.96)	156.56 (11.81)	87.83 (6.81)	169.0 (25.92)	90.54 (18.36)	62.82 (23.76)	15.84 (2.70)	66.96 (7.56)
Keidar 2013	Israel	1	18	47.7 (11.7)	9 (50)	42.5 (5.2)					119.7 (65.7)	43.6 (9.7)	88.1 (27.7)
			19	51.45 (8.3)	12 (63.2)	42 (4.8)					156.3 (75.7)	39.1 (9.9)	98.9 (29.3)
Halperin 2014	USA	1	19	50.7 (7.6)	6 (32)	36.0 (3.5)	132.8 (10.5)	81.7 (7.4)	132.3 (49.7)	154.2 (34.0)			
			19	52.6 (4.3)	9 (47)	36.5 (3.4)	126.3 (14.7)	76.6 (8.8)	162.2 (53.8)	162.5 (38.6)			
Lee 2014	Taiwan	5	30	46.4 (8.1)	8 (26.7)	31 (2.8)	128.7 (5.0)	80.1 (7.8)	230.6 (85.3)	230.6 (85.3)	262.2 (152.8)	42.8 (6.3)	142.9 (44.6)
			30	44.6 (8.6)	8 (26.7)	30.2 (2.2)	130.3 (9.3)	76.4 (8.6)	200.9 (76.6)	200.9 (76.6)	195.2 (128.3)	47.9 (9.6)	137.3 (37.8)
Parikh 2014	USA	0.5	29	46.8 (8.1)	6 (21)	32.8 (1.7)	126.4 (16.6)	77.0 (13.3)		193.4 (61.7)	196.9 (188.2)	47.2 (15.5)	101.8 (88.2)
			28	53.9 (8.4)	6 (21)	32.4 (1.8)	129.1 (19.0)	75.3 (8.1)		193.9 (66.6)	156.5 (69.1)	46.4 (13.2)	116.1 (55.9)
Ding 2015	USA	1	18	50.6 (12.6)	9 (50)	36.4 (3.0)	129 (7)	79 (5)	167 (64)	155 (34)	176 (119)	37 (9)	92 (27)
			22	51.4 (7.5)	13 (59.1)	36.7 (4.2)	126 (13)	81 (8)	155 (48)	161 (29)	145 (104)	42 (12)	91 (27)
Courcoulas 2015	USA	3	20	45.4 (7.5)	4 (20.0)	35.7 (2.7)	139.7 (12.3)	81.3 (9.6)	191.5 (82.0)	200.2 (40.3)		41.8 (8.7)	117.8 (47.5)
			20	48.9 (4.7)	3 (15.0)	35.7 (3.3)	132.0 (17.9)	76.3 (9.6)	142.1 (28.0)	182.0 (39.0)		44.1 (17.1)	105.5 (33.3)
			21	47.7 (7.0)	4 (19.0)	35.6 (3.4)	134.5 (17.0)	77.1 (8.6)	180.0 (85.4)	189.5 (55.8)		40.0 (9.3)	90.6 (49.4)
Yang 2015	China	3	32	40.4 (9.4)	9 (28.1)	31.8 (3.0)			183.6 (48.6)	90.0 (19.8)	57.6 (30.6)	19.8 (3.6)	68.4 (19.8)
			32	41.4 (9.3)	13 (40.1)	32.3 (2.4)			187.2 (39.6)	82.8 (16.2)	54.0 (36.0)	18.0 (1.8)	70.2 (16.2)
Mingrone 2015	UK	5	19	30–60	‐	44.0 (4.6)	147.5 (21.0)	92.5 (14.6)	176.4 (59.4)	84.6 (19.8)	30.6 (16.2)	19.44 (3.6)	50.4 (18.0)
			15	30–60	‐	45.4 (6.5)	157.5 (37.7)	97.3 (19.2)	178.2 (63.0)	113.4 (25.2)	46.8 (12.6)	17.64 (3.42)	73.8 (23.4)
			19	30–60	‐	44.7 (7.7)	155.3 (30.3)	96.0 (13.2)	172.8 (63.0)	100.8 (27.0)	41.4 (16.2)	17.82 (3.78)	64.8 (23.4)
Cummings 2016	USA	1	15	52.0 (8.3)	3 (20)	38.3 (3.7)	129.3 (20.6)	77.0 (10.2)	145.8 (46.8)	77.4 (18.0)	30.6 (12.6)	19.8 (5.4)	43.2 (12.6)
			17	54.6 (6.3)	10 (58.8)	37.1 (3.5)	120.1 (9.6)	74.8 (7.5)	153 (46.8)	79.2 (14.4)	41.4 (27.0)	19.8 (3.6)	39.6 (10.8)
Tang 2016	China	2	34	36.6 (8.0)	12 (35.3)	38.4 (8.6)			149.4 (39.6)	90.0 (14.4)	41.4 (36.0)	18.0 (3.6)	59.4 (14.4)
			38	40.4 (12.3)	20 (52.6)	37.8 (5.6)			162 (61.2)	93.6 (36.0)	45.0 (37.8)	18.0 (3.6)	50.4 (16.2)
Ikramuddin 2016	USA	3	60	49 (9)	22 (36.7)	34.9 (3.0)	127 (15)	78 (12)	214 (57)	182 (39)	187 (79)	41 (1)	103 (36)
			59	49 (8)	25 (42.4)	34.3 (3.1)	132 (14)	79 (10)	206 (52)	189 (46)	197 (82)	42 (9)	105 (43)
Schauer 2017	USA	5	50	48.3 (8.4)	21 (42)	37.0 (3.3)	134.7 (18.9)	81.8 (10.2)				45.8 (13.2)	100.9 (36.8)
			50	49.7 (7.4)	19 (38)	36.8 (3.0)	135.6 (17.7)	82.0 (11.4)				48.7 (12.8)	91.4 (28.9)
			50	47.9 (8.0)	11 (22)	36.2 (3.9)	136.7 (17.9)	82.2 (11.7)				44.3 (12.1)	105.7 (40.2)
Wentworth 2017	Australia	5	22	53 (6)	6 (24)	29 (1)	130 (18)	83 (10)					
			23	53 (7)	9 (35)	29 (1)	131 (11)	84 (9)					
Casajoana 2017	Spain	1	14	49.20 (9.16)	5 (35.7)	39.0 (1.68)			171.9 (64.26)				
			15	51.10 (7.70)	7 (46.7)	38.7 (2.01)			150.84 (54.00)				
			15	49.70 (8.12)	3 (47.7)	40.7 (1.34)			177.12 (94.5)				
Murphy 2018	New Zealand	1	56	46.6 (6.7)	23 (41.1)	42.5 (6.2)							
			53	45.5 (6.4)	32 (60.4)								

Abbreviations: HDL‐C, high‐density lipoprotein cholesterol; LDL‐C, low‐density lipoprotein cholesterol.

### 3.2 Inconsistency and publication bias

There was no evidence suggesting any inconsistencies between the direct and indirect network effect values in the primary outcomes (remission of diabetes for all duration and more than 3‐year follow‐up) or secondary outcomes (mean changes in glucose, systolic pressure, diastolic pressure, total cholesterol, triglycerides, HDL‐C and LDL‐C) (Figure S5A–C and E–J). However, inconsistency was only identified in the network meta‐analysis of mean weight change, and the inconsistency model was fitted in the network meta‐analysis (Figure S5D). We detected no evidence of publication bias after assessing the funnel plots (Figure S6).

### 3.3 Network meta‐analysis of primary outcomes

**FIGURE 1:**
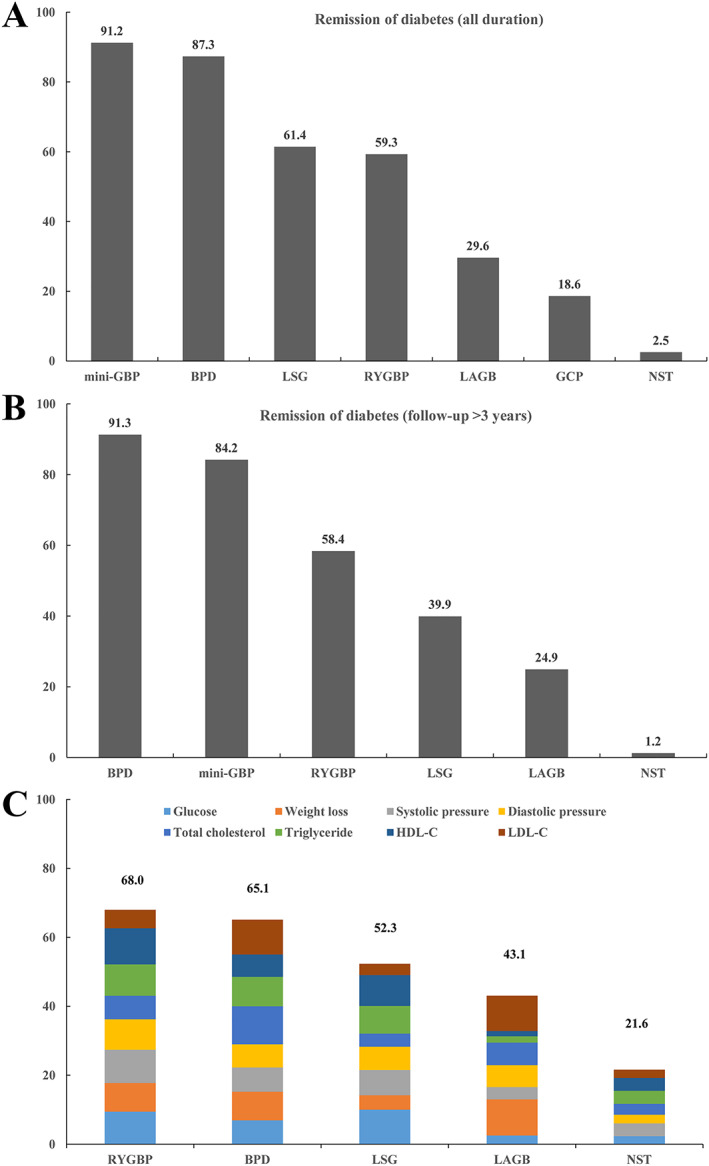
Ranking of bariatric surgeries according to primary and secondary outcomes. A, SUCRA value for remission of diabetes (all duration); B, SUCRA value for remission of diabetes (follow‐up > 3 years); C, cumulative SUCRA value after normalization for eight secondary outcomes (0–100). Every bariatric surgery was normalized with points up to a maximum of 12.5 for eight secondary outcomes, including glucose, weight loss, systolic pressure, diastolic pressure, total cholesterol, triglyceride, HDL‐C and LDL‐C, with an overall maximum score of 100. BPD, biliopancreatic diversion without duodenal switch; GCP, greater curvature plication; LAGB, laparoscopic‐adjustable gastric banding; LSG, laparoscopic sleeve gastrectomy; mini‐GBP, single anastomosis (mini) gastric bypass; NST, nonsurgical treatment; RYGBP, Roux‐en‐Y gastric bypass; SUCRA, surface under the cumulative ranking curve

For studies across short‐ and long‐term follow‐up durations, mini‐GBP (OR: 123.67, 95% CrI: 14.10, 1084.91), BPD (OR: 92.92, 95% CrI: 12.48, 691.95), LSG (OR: 24.29, 95% CrI: 8.20, 71.96), RYGBP (OR: 23.20, 95% CrI: 8.85, 60.83) and LAGB (OR: 5.57, 95% CrI: 2.32, 13.39) were all highly effective in comparison with NST at achieving the remission of diabetes, except for GCP (OR: 2.78, 95% CrI: 0.39, 19.85). BPD was more effective than GCP (OR: 33.46, 95% CrI: 2.57, 435.87) and LAGB (OR: 16.68, 95% CrI: 2.01, 138.80) at achieving remission of diabetes. GCP was found to have worse efficacy at achieving the remission of diabetes than LSG (OR: 0.11, 95% CrI: 0.02, 0.64), RYGBP (OR: 0.12, 95% CrI: 0.02, 0.67) and mini‐GBP (OR: 0.02, 95% CrI: 0.00, 0.29). LAGB was found to have better efficacy than LSG (OR: 0.23, 95% CrI: 0.07, 0.78), RYGBP (OR: 0.24, 95% CrI: 0.08, 0.74), or mini‐GBP (OR: 0.05, 95% CrI: 0.00, 0.43) at achieving the remission of diabetes (Table 2). According to the SUCRAs, the rank probability of remission of diabetes (from best to worst) among bariatric surgeries was as follows: mini‐GBP (91.2%) > BPD (87.3%) > LSG (61.4%) > RYGBP (59.3%) > LAGB (29.6%) > GCP (18.6%) > NST (2.5%) (Figure 1A).

**TABLE 2:** Network meta‐analysis for the remission of diabetes (all duration)

BPD						
**33.46 (2.57, 435.87)**	GCP					
**16.68 (2.01, 138.80)**	0.50 (0.06, 3.87)	LAGB				
3.83 (0.52, 27.87)	**0.11 (0.02, 0.64)**	**0.23 (0.07, 0.78)**	LSG			
4.01 (0.60, 26.84)	**0.12 (0.02, 0.67)**	**0.24 (0.08, 0.74)**	1.05 (0.59, 1.86)	RYGBP		
0.75 (0.05, 11.58)	**0.02 (0.00, 0.29)**	**0.05 (0.00, 0.43)**	0.20 (0.03, 1.29)	0.19 (0.03, 1.34)	Mini‐GBP	
**92.92 (12.48, 691.95)**	2.78 (0.39, 19.85)	**5.57 (2.32, 13.39)**	**24.29 (8.20, 71.96)**	**23.20 (8.85, 60.83)**	**123.67 (14.10, 1084.91)**	NST

*Note*: Comparisons between drugs should be read from left to right. The estimates are located at the crossing between the column‐defining treatment and row‐defining treatment. An OR lower than 1 favours the column‐defining treatment. The significant results are presented in bold.

Abbreviations: BPD, biliopancreatic diversion without duodenal switch; CrI, credible interval; GCP, greater curvature plication; LAGB, laparoscopic‐adjustable gastric banding; LSG, laparoscopic sleeve gastrectomy; mini‐GBP, single anastomosis (mini) gastric bypass; NST, nonsurgical treatment; OR, odds ratio; RYGBP, Roux‐en‐Y gastric bypass.

For studies with follow‐up periods greater than 3 years, BPD (OR: 54.12, 95% CrI: 7.57, 387.05), mini‐GBP (OR: 34.77, 95% CrI: 3.65, 330.70), RYGBP (OR: 10.62, 95% CrI: 2.76, 40.89) and LSG (OR: 6.83, 95% CrI: 1.47, 31.75) were significantly superior to NST in achieving the remission of diabetes, except for LAGB (OR: 3.21, 95% CrI: 0.78, 13.15). BPD was more effective at achieving remission of diabetes compared with LAGB (OR: 16.85, 95% CrI: 1.87, 152.08) and LSG (OR: 7.93, 95% CrI: 1.18, 53.28) (Table 3). According to the SUCRA, the rank probability of remission of diabetes with follow‐up period of more than 3 years (from best to worst) among bariatric surgeries: BPD (91.3%) > mini‐GBP (84.2%) > RYGBP (58.4%) > LSG (39.9%) > LAGB (24.9%) > NST (1.2%) (Figure 1B).

**TABLE 3:** Network meta‐analysis for the remission of diabetes (follow‐up > 3 years)

BPD					
**16.85 (1.87, 152.08)**	LAGB				
**7.93 (1.18, 53.28)**	0.47 (0.08, 2.68)	LSG			
5.10 (0.90, 28.75)	0.30 (0.06, 1.44)	0.64 (0.28, 1.46)	RYGBP		
1.56 (0.13, 19.32)	0.09 (0.01, 1.01)	0.20 (0.04, 1.02)	0.31 (0.05, 1.92)	Mini‐GBP	
**54.12 (7.57, 387.05)**	3.21(0.78, 13.15)	**6.83 (1.47, 31.75)**	**10.62 (2.76, 40.89)**	**34.77 (3.65, 330.73)**	NST

*Note*: Comparisons between drugs should be read from left to right. The estimates are located at the crossing between the column‐defining treatment and row‐defining treatment. An OR lower than 1 favours the column‐defining treatment. The significant results are presented in bold.

Abbreviations: BPD, biliopancreatic diversion without duodenal switch; CrI, credible interval; LAGB, laparoscopic‐adjustable gastric banding; LSG, laparoscopic sleeve gastrectomy; RYGBP, Roux‐en‐Y gastric bypass; mini‐GBP, single anastomosis (mini) gastric bypass; NST, non‐surgical treatment; OR, odds ratio.

### 3.4 Network meta‐analysis of secondary outcomes

In terms of weight loss, LSG (SMD: −2.47, 95% CrI: −4.71, −0.24) and RYGBP (SMD: −3.06, 95% CrI: −4.69, −1.42) were significantly more effective than NST. RYGBP (SMD: −1.18, 95% CrI: −2.12, −0.23) was superior to NST in lowering glucose levels. RYGBP was superior to LAGB (SMD: 2.86, 95% CrI: 0.32, 5.41) and NST (SMD: 1.93, 95% CrI: 0.51, 3.34) in controlling HDL levels. For the other secondary outcomes, including systolic and diastolic pressure, total cholesterol, triglycerides and LDL‐C, there were no significant differences among RYGBP, BPD, LSG, LAGB and NST (Table 4). According to the SUCRA, the overall rank of probability for bariatric surgeries (from best to worst) was RYGBP (68.0%) > BPD (65.1%) > LSG (52.3%) > LAGB (43.1%) > NST (21.6%) after comprehensively weighing all secondary outcomes, including glucose levels, weight loss, systolic and diastolic pressure, total cholesterol, triglycerides, HDL‐C and LDL‐C (Figure 1C).

**TABLE 4:** Network meta‐analysis of secondary outcomes

Glucose/weight loss				
BPD	2.08 (0.58, 3.58)	0.31 (−0.95, 1.57)	−0.58 (−1.75, 0.58)	−0.22 (−1.43, 0.98)
−0.76 (−2.79, 1.28)	LAGB	−0.89 (−1.98, 0.20)	−0.76 (−1.99, 0.48)	**−2.31 (−3.16, −1.47)**
0.55 (−1.52, 2.63)	1.31 (−0.20, 2.82)	LSG	0.14 (−1.00, 1.28)	**−1.42 (−2.11, −0.73)**
0.41 (−1.36, 2.18)	1.17 (−0.05, 2.39)	−0.14 (−1.26, 0.98)	RYGBP	**−1.56 (−2.46, −0.65)**
−0.77 (−2.54, 1.01)	−0.01 (−1.19, 1.18)	−1.32 (−2.73, 0.10)	**−1.18 (−2.12, −0.23)**	NST
Systolic/diastolic pressure				
BPD	−0.08 (−3.33, 3.17)	0.02 (−3.62, 3.65)	0.38 (−2.28, 3.04)	−0.82 (−3.47, 1.84)
−0.89 (−4.38, 2.59)	LAGB	0.09 (−3.13, 3.32)	0.45 (−1.68, 2.58)	−0.74 (−2.72, 1.24)
0.11 (−3.79, 4.00)	1.00 (−2.46, 4.46)	LSG	0.36 (−2.26, 2.98)	−0.83 (−3.46, 1.79)
0.51 (−2.34, 3.35)	1.40 (−0.90, 3.69)	0.40 (−2.42, 3.21)	RYGBP	−1.19 (−2.41, 0.02)
−0.73 (−3.57, 2.12)	0.17 (−1.96, 2.29)	−0.84 (−3.65, 1.98)	−1.23 (−2.54, 0.07)	NST
Total cholesterol/triglycerides				
BPD	−2.55 (−6.88, 1.78)	−0.13 (−5.42, 5.16)	−0.01 (−3.56, 3.53)	−1.65 (−5.19, 1.89)
−1.03 (−3.20, 1.15)	LAGB	2.42 (−2.46, 7.31)	2.54 (−0.37, 5.44)	0.90 (−1.75, 3.55)
−1.56 (−4.20, 1.09)	−0.53 (−2.94, 1.88)	LSG	0.11 (−3.82, 4.04)	−1.53 (−5.83, 2.78)
−1.04 (−2.83, 0.76)	−0.01 (−1.45, 1.43)	0.52 (−1.42, 2.46)	RYGBP	−1.64 (−3.40, 0.13)
−1.48 (−3.27, 0.32)	−0.45 (−1.77, 0.87)	0.08 (−2.05, 2.21)	−0.44 (−1.32, 0.45)	NST
HDL‐C/LDL‐C				
BPD	0.06 (−2.12, 2.23)	−1.26 (−3.16, 0.64)	−0.98 (−2.56, 0.60)	−1.31 (−2.89, 0.27)
1.75 (−2.08, 5.58)	LAGB	−1.32 (−3.22, 0.58)	−1.04 (−2.62, 0.54)	−1.37 (−2.96, 0.21)
−0.77 (−4.56, 3.03)	−2.52 (−5.83, 0.79)	LSG	0.28 (−0.84, 1.40)	−0.05 (−1.27, 1.17)
−1.11 (−4.22, 2.00)	**−2.86 (−5.41, −0.32)**	−0.34 (−2.65, 1.96)	RYGBP	−0.33 (−1.04, 0.38)
0.82 (−2.29, 3.93)	−0.93 (−3.29, 1.42)	1.58 (−0.90, 4.07)	**1.93 (0.51, 3.34)**	NST

*Note*: Comparisons between drugs should be read from left to right. The estimates are located at the crossing between the column‐defining treatment and row‐defining treatment. For mean change of glucose, weight loss, systolic and diastolic pressure, total cholesterol, triglycerides and LDL‐C. An SMD lower than 0.01 favours the column‐defining treatment; for mean change of HDL‐C, an SMD greater than 0.01 favours the column‐defining treatment. The significant results are presented in bold.

Abbreviations: BPD, biliopancreatic diversion without duodenal switch; CrI, credible interval; LAGB, laparoscopic‐adjustable gastric banding; LDL‐C, low‐density lipoprotein cholesterol; LSG, laparoscopic sleeve gastrectomy; HDL‐C, high‐density lipoprotein cholesterol; NST, nonsurgical treatment; RYGBP, Roux‐en‐Y gastric bypass; SMD, standardized mean difference.

## 4 DISCUSSION

Patients with morbid obesity and T2DM continue to be a major public health burden worldwide, despite some advances in its diagnosis and treatment.^15^ Recently, the prevention and treatment of diabesity—the combination of obesity and T2DM—has become an important task for physicians globally.^40^, ^41^ While sustained weight loss is highly effective in preventing and treating T2DM, it is a challenging therapeutic option for most patients. In this study, we performed a network meta‐analysis of RCTs published to date to determine the most effective bariatric surgery for patients with obesity and T2DM and obesity‐related comorbid conditions.

Our findings revealed that mini‐GBP is most likely to achieve diabetes remission. Previously, mini‐GBP was found to be an easier, safer, faster and more effective metabolic operation when compared with RYGB, which is the gold standard of bariatric surgeries.^42^, ^43^ Mini‐GBP is a new bypass procedure with a shorter operation time than RYGB and LSG, involving a single anastomosis between a long, narrow gastric pouch and an omega jejunal loop.^44^, ^45^ A landmark YOMEGA study revealed that mini‐GBP is not inferior to RYGBP on weight loss and metabolic improvement for patients with morbid obesity.^46^ Numerous studies have shown that mini‐GBP is a short and straightforward procedure leading to excellent outcomes and fewer complications, such as intestine obstruction or internal herniation, both of which are commonly associated with bariatric surgery.^47^, ^48^, ^49^ An increasing number of surgeons have adopted mini‐GBP, which has gradually become accepted as the mainstream bariatric procedure.^50^ The mechanism of glycaemic control after mini‐GBP is similar to that after RYGBP, which includes an immediate post‐operative reduction in caloric intake, durable weight loss and duodenal bypass.^51^, ^52^, ^53^ Mini‐GBP clearly combines ‘simplicity' and ‘reversibility,' two criteria of metabolic procedures proposed by the International Diabetes Federation for T2DM treatment.^54^ While SG is irreversible, RYGB is technically more challenging to perform and reverse.

Our results suggest that RYGBP is the most favourable alternative surgery to manage cardiometabolic outcomes in most patients with T2DM. RYGBP could induce weight loss with similar magnitude between adolescents and adults but higher rate of remission of diabetes and hypertension in adolescents and adults.^55^ The lipid and glucose profiles were substantially improved after RYGBP, including decreased total cholesterol, LDL‐C, triglycerides, insulin resistance (assessed by homeostasis model assessment for insulin resistance) and increased HDL‐C.^56^ In addition, RYGBP leads to significant improvements in brachial artery diameter, endothelial‐independent vasodilation and the Framingham cardiovascular risk score^57^ and offer some of the best long‐term cardiovascular benefits, especially among patients with previous risk factors.^58^ Glycated haemoglobin (HbA1c) improved more in the mini‐GBP group than in the RYGBP group for patients with type 2 diabetes, and the incidence of steatorrhea was higher in the mini‐GBP group than in the RYGBP group for the per‐protocol population.^46^, ^59^ Thus, mini‐GPB might have a similar even better effect on cardiometabolic outcomes due to their similar mechanisms of action. However, strict follow‐up after old RYGBP, as well as new mini‐GBP, is important because there is no guarantee for the same long‐term safety profile even weight loss and anti‐diabetic actions of mini‐GBP are more attractive.^59^, ^60^

From our findings, BPD appears to be most effective surgery for achieving long‐term diabetes remission in patients with obesity and T2DM. BPD was thought to be an improvement on RYGBP as it makes use of the distal 250 cm of bowel with larger gastric pouch size, an ‘eye‐ball' stomach.^61^ The long‐established BPD and its duodenal switch variant (BPD/DS) are malabsorptive procedures that have been rarely used from the past decade^62^, ^63^ due to their technical challenges and high rates of complications.^64^ Clinical data support our findings that BPD is the most effective procedure in terms of glycaemic control and weight loss. Therefore, BPD should be primarily reserved for patients with extreme obesity (BMI > 60 kg/m^2^), which requires long‐term monitoring in highly specialized medical centers.^65^

Our study assessed the bariatric surgeries currently available for treating patients with obesity and T2DM. Our network meta‐analysis incorporated all comparisons of available bariatric surgeries into a single analysis. In addition, cardiometabolic outcomes were included in our analysis to provide a ranking of available bariatric surgeries when different cardiometabolic outcomes were taken into consideration. However, no evidence was available to determine the effect of mini‐GBP on cardiometabolic outcomes. The short‐ and long‐term effects of bariatric surgery in the control of diabetes and management of cardiometabolic conditions suggest that the manipulation of the stomach or intestines through surgery, medical devices, or drugs may be the most radical change in the treatment of T2DM in the past century.^51^

The present study should be interpreted with caution in view of the following limitations. First, a limited number of trials were included in this study. On the one hand, only a few RCTs on mini‐GBP and BPD have been reported in the literature. On the other hand, cardiometabolic data were not available for mini‐GBP. Second, inconsistencies were identified for the network meta‐analysis of weight loss. Third, studies with subgroups of patients with obesity and T2DM were not included in this study, as these patients were not randomized. Fourth, none of the three bariatric surgeries, including mini‐GBP, BPD and RYGBP, showed significant difference with LSG in terms of diabetes remission regardless of time period, long‐term diabetes remission and eight cardiometabolic conditions, respectively. Finally, variations in the follow‐up periods may have affected the OR outcome measures. However, we attempted to overcome these limitations by conducting subgroup analyses for studies with more than 3‐year follow‐up periods, which were consistent with the overall analysis.

## 5 CONCLUSION

In summary, mini‐GBP is more likely to achieve diabetes remission when compared with other bariatric surgeries, but BPD appears to be the most effective surgery for achieving long‐term diabetes remission. RYGBP is the most favourable alternative to manage cardiometabolic conditions. The effects of mini‐GBP on cardiometabolic outcomes were inconclusive and require future studies.

## CONFLICT OF INTEREST

The authors confirm that there are no conflicts of interest.

## Supporting information


**Figure S1**. Flow chart of literature search.
**Figure S2.** Risk of bias graph.
**Figure S3.** Risk of bias summary figure.
**Figure S4.** Network plot of eligible comparisons for the included randomized trials in terms of primary and secondary outcomes.
**Figure S5.** Inconsistency plot for eligible comparisons for the included randomized trials in terms of primary and secondary outcomes.
**Figure S6.** Funnel plot for eligible comparisons for the included randomized trials in terms of primary and secondary outcomes
