# Supplementary material for: Comparative effectiveness of bariatric surgeries in patients with obesity and type 2 diabetes mellitus: A network meta‐analysis of randomized controlled trials
Source: Obes Rev. 2020 Apr 14;21(8):e13030. doi: 10.1111/obr.13030 (PMC7379237; doi:10.1111/obr.13030)
Supplement: Supplementary file 1 — Figure S1. Flow chart of literature search. Figure S2. Risk of bias graph. Figure S3. Risk of bias summary figure. Figure S4. Network plot of eligible comparisons for the included randomized trials in terms of primary and secondary outcomes. Figure S5. Inconsistency plot for eligible comparisons for the included randomized trials in terms of primary and secondary outcomes. Figure S6. Funnel plot for eligible comparisons for the included randomized trials in terms of primary and secondary outcomes [file OBR-21-e13030-s001.docx]

# **Supplementary Material**

**Title**: Comparative effectiveness of bariatric surgeries in patients with obesity and type 2 diabetes mellitus: a network meta-analysis of randomized controlled trials

**Journal:** Obesity Reviews

**Authors:** Li Ding, Yuxin Fan, Hui Li, Yalan Zhang, Dongwang Qi, Shaofang Tang, Jingqiu Cui, Qing He, Chuanjun Zhuo, Ming Liu

**Corresponding author**: Chuanjun Zhuo, Ming Liu

**Table of content**

**sFigure 1**. Flow chart of literature search.

**sFigure 2.** Risk of bias graph.

**sFigure 3.** Risk of bias summary figure.

**sFigure 4.** Network plot of eligible comparisons for the included randomized trials in terms of primary and secondary outcomes.

**sFigure 5.** Inconsistency plot for eligible comparisons for the included randomized trials in terms of primary and secondary outcomes.

**sFigure 6.** Funnel plot for eligible comparisons for the included randomized trials in terms of primary and secondary outcomes


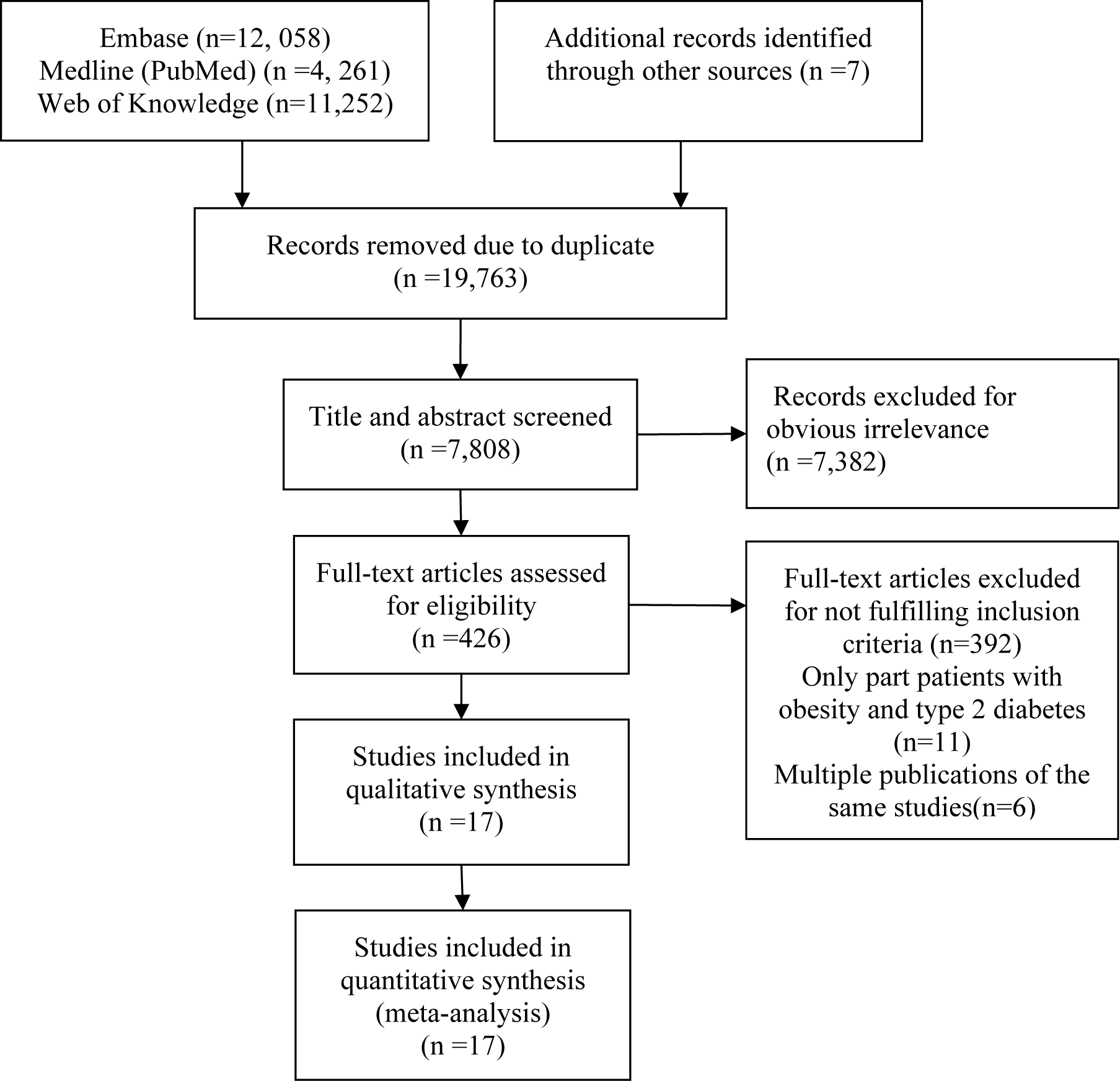


**sFigure 1**. Flow chart of literature search.

**
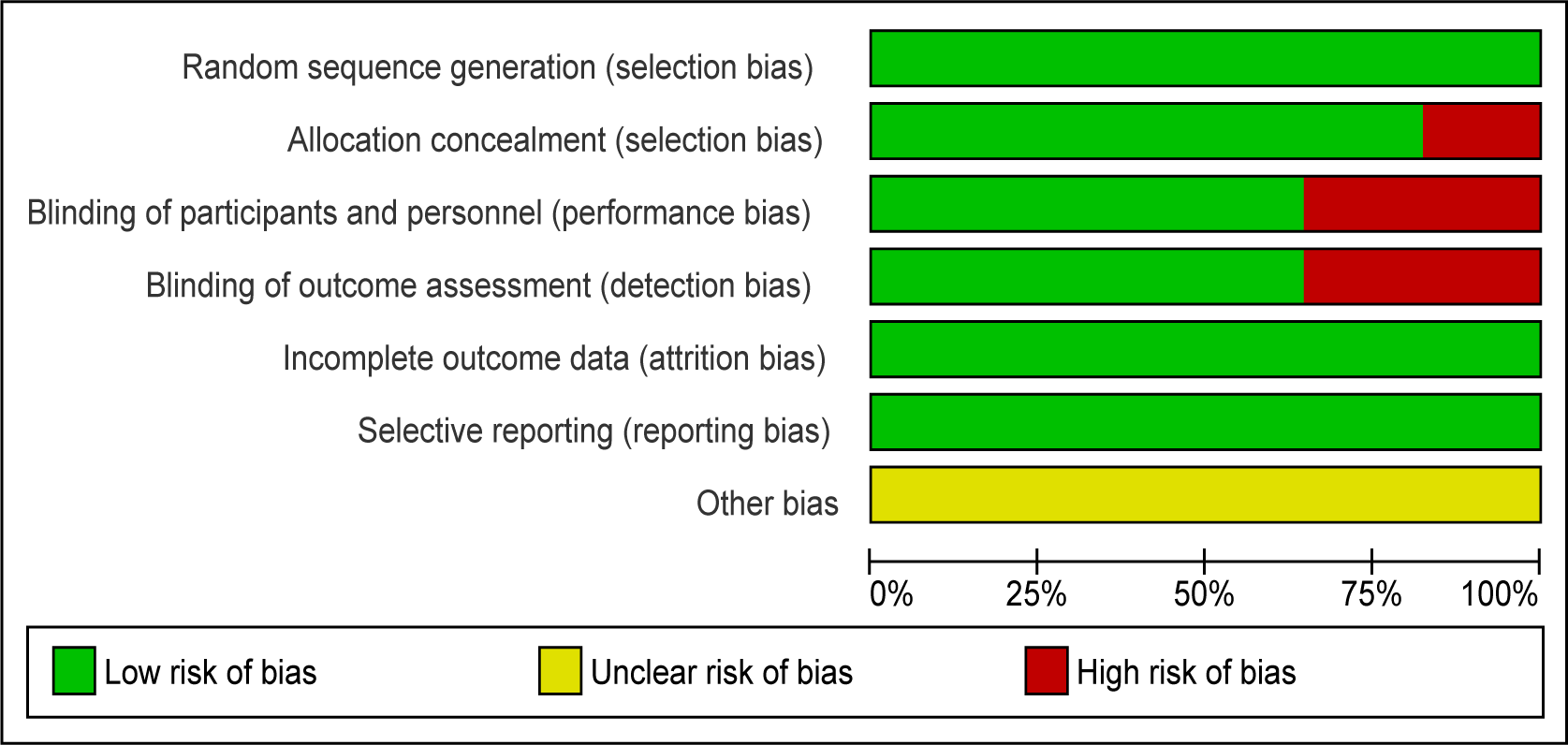
**

**sFigure 2.** Risk of bias graph.


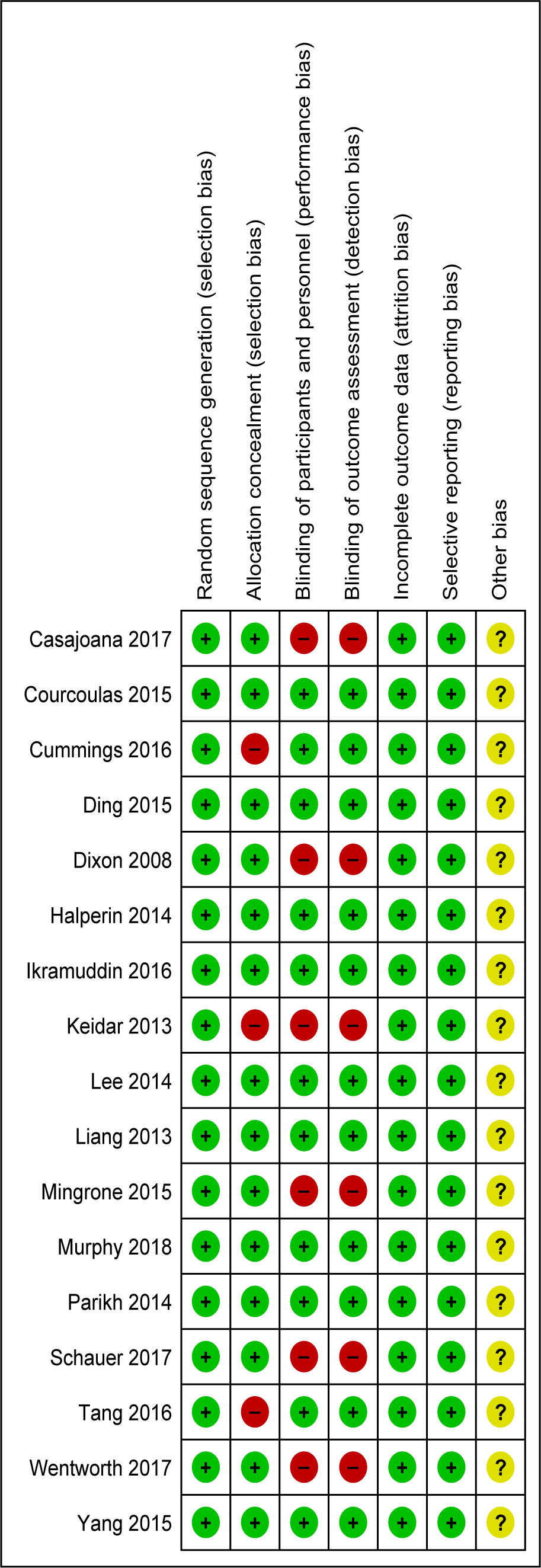


**sFigure 3.** Risk of bias summary figure.

**
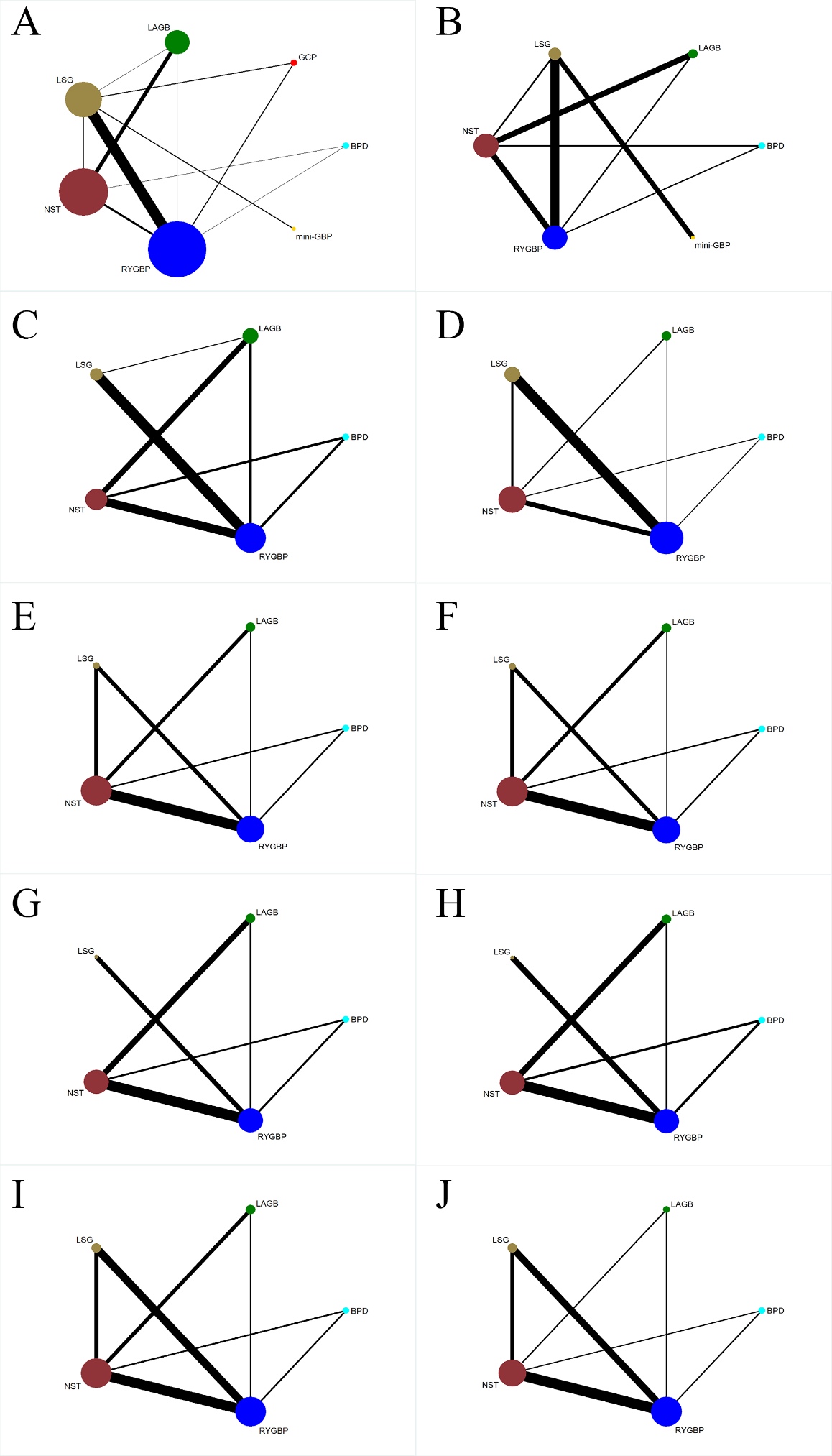
**

**sFigure 4**. Network plot of eligible comparisons for the included randomized trials in terms of primary and secondary outcomes. (A) Remission of diabetes (all duration), (B) remission of diabetes (follow-up >3 years), (C) mean change of glucose, (D) mean change of weight loss, (E) mean change of systolic pressure, (F) mean change of diastolic pressure, (G) mean change of total cholesterol, (H) mean change of triglycerides, (I)mean change of HDL-C, and (J) mean change of LDL-C.

**
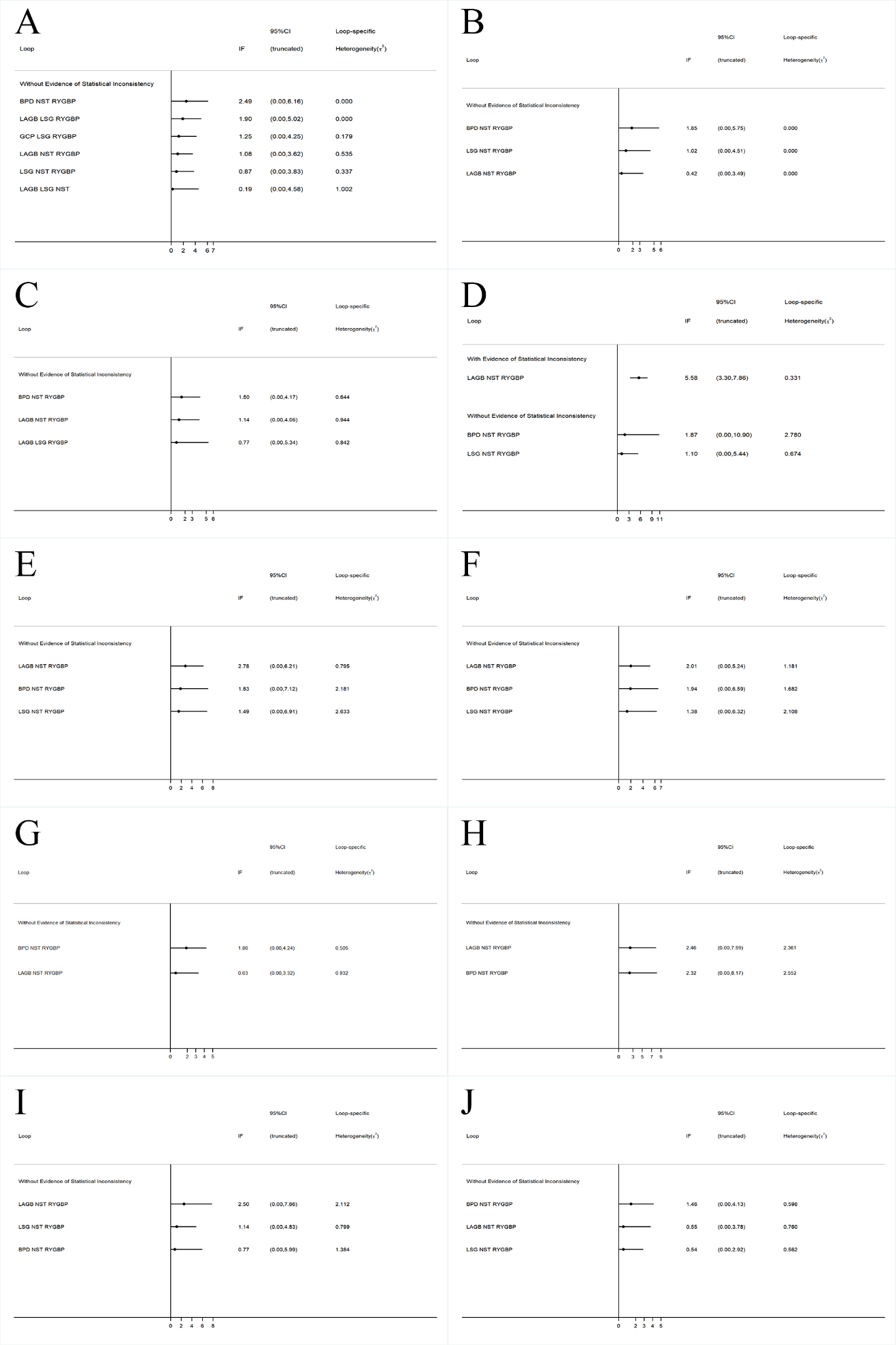
**

**sFigure 5**. Inconsistency plot for eligible comparisons for the included randomized trials in terms of primary and secondary outcomes. (A) Remission of diabetes (all duration), (B) remission of diabetes (follow-up >3 years), (C) mean change of glucose, (D) mean change of weight loss, (E) mean change of systolic pressure, (F) mean change of diastolic pressure, (G) mean change of total cholesterol, (H) mean change of triglycerides, (I)mean change of HDL-C, and (J) mean change of LDL-C.

**
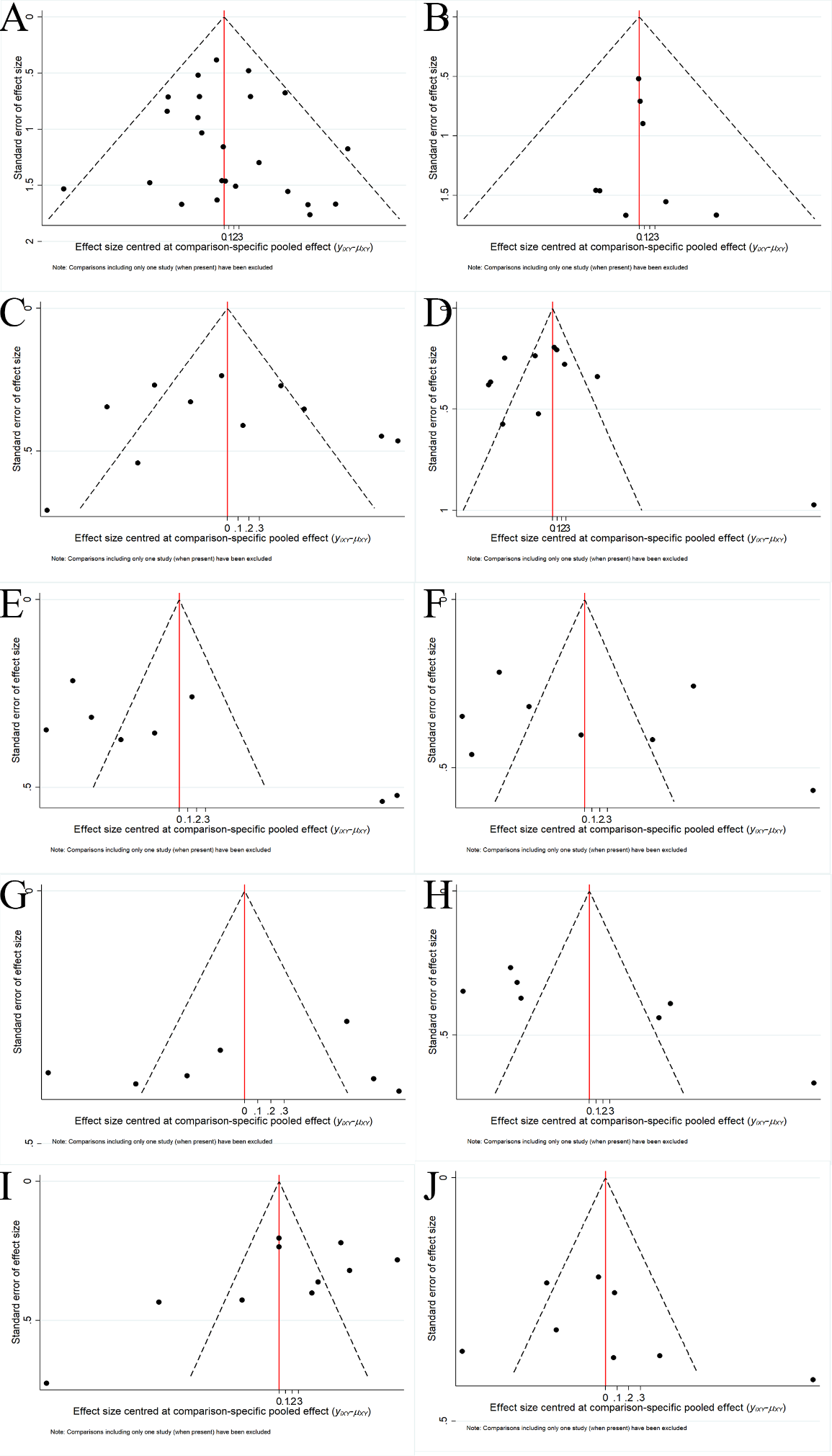
**

**sFigure 6**. Funnel plot for eligible comparisons for the included randomized trials in terms of primary and secondary outcomes. (A) Remission of diabetes (all duration), (B) remission of diabetes (follow-up >3 years), (C) mean change of glucose, (D) mean change of weight loss, (E) mean change of systolic pressure, (F) mean change of diastolic pressure, (G) mean change of total cholesterol, (H) mean change of triglycerides, (I) mean change of HDL-C, and (J) mean change of LDL-C.
